# Effects of Online Pilates and Face-to-Face Pilates Intervention on Body Composition, Muscle Mechanical Properties, Cardiometabolic Parameters, Mental Health, and Physical Fitness in Middle-Aged Women with Obesity

**DOI:** 10.3390/healthcare11202768

**Published:** 2023-10-19

**Authors:** Hun-Young Park, Kyounghwa Jung, Won-Sang Jung, Sung-Woo Kim, Jisu Kim, Kiwon Lim

**Affiliations:** 1 Department of Sports Medicine and Science, Graduate School, Konkuk University, 120 Neungdong-ro, Gwangjin-gu, Seoul 05029, Republic of Korea; parkhy1980@konkuk.ac.kr (H.-Y.P.); kswrha@konkuk.ac.kr (S.-W.K.); kimpro@konkuk.ac.kr (J.K.); 2 Physical Activity and Performance Institute, Konkuk University, 120 Neungdong-ro, Gwangjin-gu, Seoul 05029, Republic of Korea; pilateslab@konkuk.ac.kr; 3 Department of Physical Education, Konkuk University, Seoul 05029, Republic of Korea; 4 Department of Senior Exercise Prescription, Dongseo University, Busan 47011, Republic of Korea; jws1197@dongseo.ac.kr

**Keywords:** pilates, health-related function, obese women, COVID-19

## Abstract

With the emergence of coronavirus disease 2019, individuals have been participating in online exercises to maintain their health while avoiding infection. Among these online exercises, Pilates intervention is a popular modality. This study aimed to examine the differences between online and face-to-face Pilates interventions in terms of various physiological parameters and included 30 middle-aged individuals (age 43.3 ± 5.5 years) with obesity. These individuals were randomly divided into a face-to-face Pilates group (FPG), an online Pilates group (OPG), and a control group (CG). The FPG and OPG performed a 60-min mat Pilates program with a Borg scale of 11–17, three times a week for 12 weeks. The participants in the CG maintained their daily routines. Body composition, mechanical muscle properties, cardiometabolic parameters, mental health, and physical fitness were assessed before and after 12 weeks of intervention. No significant differences in body composition or cardiometabolic parameters were observed between groups. However, the FPG and OPG showed greater improvements than the CG in terms of muscle mechanical properties, cardiometabolic parameters, mental health, and physical fitness. In addition, the FPG showed greater improvement than the OPG. In conclusion, face-to-face Pilates is a more effective modality than online Pilates, although both modalities improve health-related parameters.

## 1. Introduction

Social changes caused by coronavirus disease 2019 (COVID-19) resulted in restrictions on physical activity and changes in exercise methods; social distancing to minimize the route of infection has reduced physical activity, altered eating habits, and intensified emotional problems [1]. In this situation, online programs have been used as alternatives to in-person physical activity; even now, when the COVID-19 pandemic has ended, various methods to increase physical activity levels are being applied [1,2,3].

Obesity is a global health problem caused by excessive body fat accumulation and has been reported to increase the prevalence and risk of death as a major cause of various metabolic and cardiovascular diseases [4]. Physical activity lowers overall body fat levels by reducing subcutaneous and visceral fat and is very effective in treating obesity [5,6,7]. The most common exercise method used for the treatment of obesity is aerobic exercise, in which moderate-intensity exercise is performed using a treadmill or bicycle continuously without rest for a long time of at least 30 min [4]. However, because this type of aerobic exercise increases the risk of musculoskeletal damage by placing a burden on the joints of individuals with obesity who have difficulty supporting their own weight owing to excessive weight [8], a new exercise treatment is needed. Accordingly, the effectiveness of Pilates, a combined exercise treatment for people with obesity, has been suggested in various studies [9,10,11,12], and the American College of Sports Medicine recommends Pilates for obesity and health promotion [13].

Pilates is widely used as an exercise modality to increase the amount of physical activity within the limited environmental conditions of an online platform, as well as in a face-to-face environment [14,15,16]. Pilates is a low-to-moderate-intensity exercise that can be easily performed by various individuals, has fewer spatial restrictions, corrects incorrect postures through various movements, and improves body control [8,9,17,18]. In addition, Pilates is effective in improving health status and preventing diseases by enhancing metabolism and cardiovascular function [9,11,12].

However, online exercise intervention has mixed positive and negative aspects due to the low exercise participation rate and differences in exercise time, intensity, and frequency compared with those of face-to-face exercise intervention [19,20,21,22,23]. Suner-Keklik et al. [23] reported that an online mat Pilates intervention for six weeks improved core muscles and proprioceptive function in healthy adult women. Fleming et al. [24] reported that online Pilates exercise intervention for 8 weeks reduced pain, anxiety, and depression in patients with multiple sclerosis. Bulguroglu and Bulguroglu [12] indicated that eight weeks of online and face-to-face Pilates intervention showed similar benefits on core muscular endurance, depression, and quality of life in healthy individuals. Meanwhile, Curtis et al. [19] reported that the effect of online exercise intervention was not clear because it showed a shorter exercise time and lower exercise participation and continuity rates than face-to-face exercise intervention. Janjua et al. [20] argued that exercise intervention using an online platform, based on a systematic review, had a high dropout rate of participants and low statistical power compared with face-to-face exercise intervention; therefore, no clear advantage was identified.

Very few studies have verified the effectiveness of face-to-face exercise, which is generally used today, and online exercise, which is highly time-efficient and based on the same exercise intervention program (e.g., same exercise volume, intensity, time, etc.). A few studies using online platforms have reported that Pilates intervention is effective in improving body composition, core muscle stability, and mental health [12,19,20,24]. However, to increase the reliability of the effectiveness of online Pilates intervention, it is necessary to verify its effectiveness in health promotion and disease improvement compared to face-to-face Pilates intervention. In particular, studies that have comprehensively examined the differences in the effects of face-to-face and online Pilates interventions on body composition, core muscle mechanical properties, cardiometabolic parameters, mental health, and physical fitness factors in women with obesity are lacking. Therefore, it is important to confirm the effectiveness of online and face-to-face Pilates exercises using the same exercise treatment.

This study aimed to examine the effects of face-to-face and online Pilates interventions for 12 weeks on body composition, core muscle mechanical properties, cardiometabolic function, mental health, and physical fitness in middle-aged women with obesity. Our hypothesis was that face-to-face Pilates intervention, with more accurate exercise feedback from instructors, may result in greater enhancement of body composition, core muscle mechanical properties, cardiometabolic parameters, mental health, and physical fitness factors than online Pilates intervention in women with obesity.

## 2. Materials and Methods

### 2.1. Participants

We selected 45 premenopausal middle-aged women with obesity (>30% body fat) aged 30–50 years, who were nonsmokers and had no history of musculoskeletal, cardiovascular, or pulmonary diseases. All participants received information regarding the purpose and process of the study and provided informed consent after receiving sufficient explanation regarding the experiment and possible adverse effects prior to the start of the study. They were then randomly divided into three groups using a computerized random sampling generator: FPG (*n* = 15), online Pilates group OPG (*n* = 15), and control group CG (*n* = 15). However, 15 participants (FPG,5; OPG,5; CG,5) were excluded from the study due to withdrawal (*n* = 8) and discontinuation of the intervention (*n* = 7). Therefore, 30 participants (10 in each group) completed the study, and the data were analyzed.

The sample size was selected as follows in line with the intention to improve the test power to 95% or more: based on the serum triglyceride (TG) mean and standard deviation from the Nahm and Shin [25] study, the effect size was calculated using the method presented by Cohen [26], and the result was 0.598, and α = 0.05, 1-β (power) was set to 0.8; the number of groups was 3, and the number of repeated measurements was set to 2; and as a result of calculating G-power, the sample size was 30 people. However, considering the dropout rate of a study using the online platform proposed by Curtis et al. [19], 15 participants were selected from each group.

**Figure 1:**
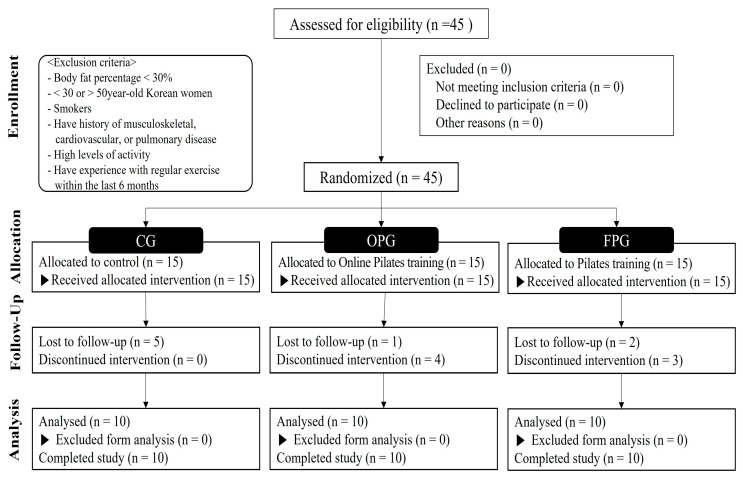
The consolidated standards of reporting trial flow diagram. CG = control group, OPG = online Pilates group, FPG = face-to-face Pilates group.

The consolidated standards of the reporting trial flow diagram are shown in Figure 1, and the characteristics of the participants are presented in Table 1. This trial was reviewed and approved by the Institutional Review Board of Konkuk University (7001355-202112-HR-496) and was conducted in accordance with the Declaration of Helsinki. The trial information was registered with the Clinical Research Information Service of Korea (KCT0008469).

### 2.2. Study Design

The design for this study was as follows: a pre-intervention session, followed by a 12-week intervention, and a post-intervention session.

In the pre- and post-intervention sessions, all participants fasted for >8 h. After stabilization, a fingertip blood sample was collected between 7:00 and 9:00 a.m. for the analysis of cardiometabolic biomarkers. After a 30-min recovery, blood pressure, mental health parameters, anthropometry, body composition, and muscle mechanical properties were measured. After sufficient rest and meals, physical fitness-related parameters were measured.

During the 12-week intervention session, the FPG performed a face-to-face mat Pilates intervention with the instructor, the OPG performed an online Pilates intervention based on a video preproduced in advance, and the CG was encouraged to maintain normal daily routines during the intervention period.

**Figure 2:**
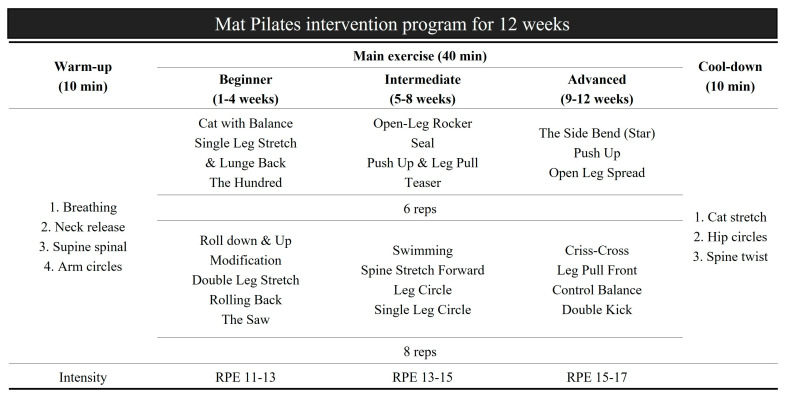
Pilates intervention program for FPG and OPG. FPG = face-to-face Pilates group, OPG = online Pilates group, reps = repetitions, RPE = rating of perceived exertion.

The FPG and OPG performed a 60-min mat Pilates intervention program three times a week for 12 weeks. The exercise intensity was gradually increased at intervals of 4 weeks, and three difficulty levels were configured. The mat Pilates intervention program is shown in detail in Figure 2. Both groups were instructed by an instructor on the correct movements and precautions of Pilates before the mat Pilates intervention. The Pilates intervention program selected mat Pilates movements suitable for middle-aged women, and the exercise stage was divided into beginner (11–13), intermediate (13–15), and advanced (15–17) according to the rating of perceived exertion (RPE) using a 20 Borg scale [27]. The Pilates intervention for the FPG and OPG consisted of 10-min of warm-up, 40-min of the main exercise, and 10-min of cool-down.

In the present study, to increase compliance with Pilates intervention for the participants in the OPG, we confirmed Pilates intervention participation using wired messages and asked the participants to take and submit Pilates intervention diaries, photos, and videos. In addition, we conducted training on precise movements and precautions before sharing new Pilates intervention program videos updated every four weeks with the participants, and interviews were conducted every month to improve compliance with the Pilates intervention.

### 2.3. Outcome Measurements

#### 2.3.1. Anthropometry and Body Composition

Anthropometry and body composition parameters (e.g., height, weight, body mass index [BMI], free fat mass [FFM], skeletal muscle mass [SMM], fat mass [FM], percent body fat [% BF], and waist–hip ratio [WHR]) of all participants were analyzed using a stadiometer (YM-1, KDS, Seoul, Republic of Korea) and Dual-energy X-ray absorptiometry (PRIMUS, Osteosys, Seoul, Republic of Korea). All participants wore lightweight clothing and any metal items on their bodies were discarded.

#### 2.3.2. Muscle Mechanical Property

The mechanical properties of the trunk muscles (e.g., muscle tone and stiffness) were measured using a Myoton PRO (Myoton AS, Tallinn, Estonia), which is a contact-type soft tissue measuring device with a built-in 3-axis digital acceleration sensor. Myoton PRO (Myoton AS, Tallinn, Estonia). Myoton PRO is a device whose reliability and validity have been proven for muscle tone evaluation [28]; the muscle tone and stiffness of the rectus abdominis, external oblique, and erector spinae muscles were measured using this device.

#### 2.3.3. Cardiometabolic Parameters

The resting blood pressure was measured using an automatic sphygmomanometer (ACCUNIQ BP210; SELVAS Healthcare, Daejeon, Republic of Korea). After the participants sat on a chair and rested for at least 5 min using the automatic pressurization method, automatic pressurization was performed for 20 s in a comfortable sitting posture with the right upper arm at the same level as the heart. Systolic blood pressure (SBP) and diastolic blood pressure (DBP) were measured twice, and the average values were used. The mean arterial pressure (MAP) was calculated using the following equation:

MAP = (SBP − DBP)/3 + DBP

Blood lipid concentrations and blood glucose levels were analyzed using a Lipidocare analyzer (Standard Lipidocare, SD BIOSENSOR, Suwon, Republic of Korea). Blood was collected using the fingertip method after fasting for more than 8 h before the test. Blood lipid concentrations were measured using lipid profile strips to determine triglyceride (TG), total cholesterol (TC), high-density lipoprotein cholesterol (HDL-C), and low-density lipoprotein cholesterol (LDL-C) levels, and blood glucose was measured using a glucose strip.

#### 2.3.4. Mental Health

To analyze the changes in autonomic nervous system (ANS) activity, heart rate variability (HRV), which quantifies the degree of physiological heart rate fluctuations, was measured without taking drugs that could affect the ANS. After having the participants sit comfortably, a Polar V800 (Polar Electro Oy, Kempele, Finland) was worn on the left and right sides below the clavicle and below the 10th rib, and after resting for 10 min, measurements were taken for 5 min thereafter. ANS activity was evaluated by dividing HRV into time and frequency domains based on the standard values of the European Society of Cardiology and North American Society of Cardiology Electrophysiology. The analyzed data were in the time domain (mean RR, standard deviation of the interval; SDNN, the square root of the mean of the sum of the squares of differences between adjacent NN intervals; RMSSD) and frequency domain (low frequency; LF, high frequency; HF, and LF/HF ratio).

The State-Trait Anxiety Inventory (STAI) used in the present study has been used clinically as a tool to measure the anxiety state of normal adults without mental disorders [29]. The STAI consists of 20 questions about expressing the respondent’s feelings and emotions or about the individual’s innate emotional anxiety characteristics and presents options on a 4-point scale. The results of the scale are structured such that a minimum score of 20 points and a maximum score of 80 points can be obtained.

An experiment was conducted using the Beck Depression Inventory (BDI) scale as a scale to evaluate depression levels [30]. It consists of 21 questions covering the cognitive, emotional, motivational, and physical symptom domains of depression. The criteria for the BDI score are as follows: a score of 0–10 is normal, a score of 11–16 indicates a mild mood disorder, a score of 17–20 indicates clinical borderline depressive disorder, a score of 21–30 indicates moderate depression, a score of 31–40 indicates severe depression, and a score of 40 indicates severe depression.

#### 2.3.5. Physical Fitness

Grip strength was evaluated by measuring the maximum muscle strength of the forearm. The participants stood with their feet shoulder-width apart and their arms naturally lowered. A digital dynamometer (TKK 5401; Takei, Tokyo, Japan) was used to adjust the handle such that the second knuckle of the index finger was positioned at a right angle. The participant then straightened their arms, maintained a 15° distance between the torso and arms, and provided maximum force for 2–3 s. The left and right sides were measured twice and the values were recorded to the first decimal place in units of 0.1 kg.

The situations were measured to evaluate muscular endurance. The participants laid on their backs, bent their legs at an angle of approximately 70° to 90°, and fixed their toes. Both hands were crossed and placed on the shoulders, and only motions in which both elbows touched the legs were counted. The time limit was set to 1 min, along with the opening slogan, and the number of repetitions was recorded.

Flexibility was measured by sitting forward and bending (cm) using a left forward flexion tester (T.K.K. 5403, Flexion-D, Tokyo, Japan). After the participant fully adhered the soles of their feet to the vertical surface of the measuring instrument, without bending their knees, they slowly bent their upper body when signaled to start, such that the middle fingers of both hands touched the measuring instrument simultaneously. The participant pushed forward as much as possible and measured while stopped for more than 3 s. If the correct posture was not maintained, it was judged invalid, and after measuring a total of two times, the higher value was recorded in 0.1 cm increments.

A one-leg stand with the eyes closed was used to evaluate static balance. In the one-leg stand with eyes closed, participants were asked to maintain balance by keeping both hands on their waist while standing naturally with both eyes open and lifting one foot; the record was measured when the eyes were closed. If the supporting foot moved or the hand attached to the waist fell, it was considered a loss of balance, and the time was recorded.

To evaluate cardiorespiratory endurance, the maximum oxygen uptake (VO_2_max) was measured using a ramp exercise protocol with a bicycle ergometer (Aerobike 75XLIII, Konami, Tokyo, Japan) [9]. First, after attaching the heart rate sensor to the earlobe, each participant’s information (e.g., height, weight, and age) was entered. Then, after resting until the heart rate stabilized, the exercise was performed. During exercise, pedal exercise was performed at a speed that maintained the number of pedals at 50 rpm for 1 min, and exercise was performed continuously until the heart rate reached 75% of the maximal heart rate (HRmax) (female HRmax: 205 − 0.75 × age) using a ramp protocol (15 W/min). The VO_2_max was calculated using a regression equation (oxygen uptake = 9.386 watt + 289.6).

### 2.4. Statistical Analysis

The means and standard deviations were calculated for each primary outcome parameter. The normality of the distribution of all outcome parameters was verified using the Shapiro–Wilk W-test prior to the parametric tests. A two-way analysis of variance (time × group) with repeated measures of the time factor was used to analyze the effects of the Pilates intervention on each outcome parameter. Partial eta-squared (*η*^2^) values were calculated as measures of effect size for the analysis of variance. If a significant interaction or main effect within time was found, a Bonferroni post hoc test was performed to identify within-group changes over time. Additionally, a paired *t*-test was performed to compare the post-intervention versus pre-intervention values of the outcome parameters in each group. All analyses were performed using SPSS Statistics (version 25.0; IBM Corp., Armonk, NY, USA).

## 3. Results

### 3.1. Anthropometry and Body Composition

The anthropometric and body composition data of all groups before and after the intervention are shown in Table 2. No significant interaction or main effect within time was observed for any of the anthropometric or body composition parameters. In other words, there was no change in anthropometry or body composition after face-to-face and online Pilates interventions for 12 weeks.

### 3.2. Muscle Mechanical Property

Table 3 shows the pre- and post-intervention mechanical muscle data. There was a significant interaction and main effect within time in muscle tone and stiffness (*η*^2^ > 0.225, *p* < 0.05) of all core muscles (e.g., the rectus abdominis, external oblique, and erector spinae). Post hoc analysis revealed a significant decrease in the muscle tone and stiffness of the rectus abdominis (tone: *p* < 0.01, stiffness: *p* < 0.05), external oblique (tone: *p* < 0.001, stiffness: *p* < 0.01), and erector spinae (tone: *p* < 0.01, stiffness: *p* < 0.01) muscles in the FPG. In addition, there was a significant decrease in the muscle tone and stiffness of the rectus abdominis (tone, *p* < 0.05; stiffness, *p* < 0.05), external oblique (tone, *p* < 0.05; stiffness, *p* < 0.05), and erector spinae (tone, *p* < 0.05; stiffness, *p* < 0.05) in the OPG. However, improvements in core muscle mechanical properties (e.g., decreased muscle tone and stiffness) were greater with the FPG than with the OPG.

### 3.3. Cardiometabolic Parameters

The pre- and post-intervention data for the cardiometabolic parameters in all groups are shown in Table 4. No significant interaction or main effect within time was observed for any of the cardiometabolic parameters. In other words, there was no change in the cardiometabolic parameters after face-to-face and online Pilates interventions for 12 weeks.

### 3.4. Mental Health

Table 5 presents the pre- and post-intervention mental health data. There was a significant interaction between SDNN (*η*^2^ = 0.326, *p* = 0.005), RMSSD (*η*^2^ = 0.261, *p* = 0.017), and LF/HF ratio (*η*^2^ = 0.210, *p* = 0.041), and a significant main effect within time was found in the mean RR (*η*^2^ = 0.174, *p* = 0.024). In addition, STAI (*η*^2^ = 0.250, *p* = 0.020) and BDI (*η*^2^ = 0.204, *p* = 0.046) scores showed a significant interaction. Post hoc analysis revealed a significant increase in mean RR (*p* < 0.05), SDNN (*p* < 0.001) and RMSSD (*p* < 0.05), and a significant decrease in LF/HF ratio (*p* < 0.05), STAI (*p* < 0.01), and BDI (*p* < 0.05) in the FPG. In addition, there was a significant increase in the RMSSD (*p* < 0.05) and a significant decrease in the LF/HF ratio (*p* < 0.05) and STAI (*p* < 0.05). However, improvements in mental health parameters were greater with the FPG than with the OPG.

### 3.5. Physical Fitness

The physical fitness data of all groups before and after the intervention are presented in Table 6. There was a significant interaction between sit-and-reach (*η*^2^ = 0.322, *p* = 0.005), and a significant main effect within time in situ (*η*^2^ = 0.360, *p* = 0.001), right balance (*η*^2^ = 0.235, *p* = 0.008), and left balance (*η*^2^ = 0.203, *p* = 0.014). Post hoc analysis revealed a significant increase in sit-ups (*p* < 0.01), sit-and-reach (*p* < 0.01), right balance (*p* < 0.05), and left balance (*p* < 0.05) in the FPG. In addition, there was a significant increase in the sit-up (*p* < 0.05) and sit-and-reach (*p* < 0.05) movements in the OPG. However, improvements in these parameters (e.g., sit-and-reach, sit-up, right balance, and left balance) were greater in the FPG than in the OPG.

## 4. Discussion

According to the hypothesis of the present study, face-to-face Pilates intervention based on more accurate exercise participation by an instructor induces greater improvements in body composition, core muscle mechanical properties, cardiometabolic parameters, mental health, and physical fitness factors than online Pilates intervention in women with obesity. Consistent with this, the FPG and OPG showed significant improvements in core muscle mechanical properties, mental health, and physical fitness factors compared to the CG, and the FPG showed greater enhancement than the OPG. However, the Pilates intervention had no effect on the body composition and cardiometabolic parameters of the FPG and OPG in women with obesity.

Previous studies have reported various results related to changes in body composition following Pilates. Segel et al. [31] showed that a 24-week Pilates intervention (once a week and for 1 h a day) did not induce significant changes in healthy middle-aged adults; they argued that Pilates, which consists of isometric and core-centered exercises, is more effective in improving the balance and stability of core muscles than in reducing fat mass and increasing muscle mass. De Souza Cavina et al. [32] determined the efficacy of mat Pilates intervention on body composition in healthy adults compared with that of traditional exercise or control condition models using a systematic review and meta-analysis and concluded that mat Pilates intervention is no better than the control condition or other exercise modalities to improve body composition due to lower exercise intensity and exercise volume. Aibar-Almazán et al. [33] evaluated the effect of a 12-week Pilates intervention on body composition parameters (e.g., BMI, percentage of body fat, and SMM) after participants were randomly allocated to either a control (*n* = 54) or a Pilates (*n* = 55) group among community-dwelling Spanish women aged 60 years and older. They concluded that the 12-week Pilates intervention showed beneficial effects on BMI but failed to induce changes in other body compositions. In contrast, Avkin and Aslan [34] confirmed the effect of an 8-week Pilates intervention performed with 11–17 RPE for 90 min three times a week on body composition in sedentary women with overweight and obesity, concluding that 8-week Pilates exercises have positive effects on body composition (e.g., weight, BMI, percent body fat, waist, abdomen, and hip circumference) in the cohort under investigation. In the present study, we applied a 60-min Pilates intervention with 11–17 RPE (three times a week for 12 weeks) to middle-aged women with obesity, as at least 150–300 min of moderate-intensity physical activity per week is generally recommended for weight loss [9,35,36]. Nevertheless, the present study did not confirm any positive changes following Pilates intervention in middle-aged women with obesity. Our results are consistent with those of Jung et al. [9], who reported that mat Pilates does not have a positive effect on body composition and cardiometabolic parameters because it involves low exercise intensity and exercise volume according to heart rate and energy expenditure. Therefore, we believe that higher exercise intensity, time, and frequency are needed to induce positive changes in body composition via a Pilates intervention.

Consistent with the body composition results, no positive changes were observed in cardiometabolic parameters owing to our face-to-face and online Pilates interventions in middle-aged women with obesity. A few previous studies [37,38,39] have reported that Pilates intervention has a positive effect on cardiometabolic parameters; however, other studies [9,40,41] have reported that it is not a useful strategy for positive changes in cardiometabolic parameters. Kim et al. [38] verified the effects of an 8-week Pilates intervention on lipid metabolism and inflammatory cytokine mRNA expression in female undergraduates in their 20 s who had no prior experience in Pilates intervention and had not exercised in the previous 6 months. They concluded that an 8-week Pilates intervention had no effect on TG, TC, and LDL-C but had a positive effect on creatine kinase expression and HDL-C. Chen et al. [37] performed a systematic review and meta-analysis to assess the effects of Pilates on blood glucose and lipid levels. They concluded that the Pilates intervention could improve postprandial blood glucose, fasting blood glucose, glycosylated hemoglobin, TG, TC, and LDL-C levels in patients with diabetes, which could be influenced by its duration and intensity. However, it had no significant effect on blood glucose or lipid levels in non-diabetic individuals. Khajehlandi and Mohammadi [39] reported that a 12-week Pilates intervention (60 min per day, 3 times a week) resulted in significant improvements in BMI, TC, TG, and HDL-C in sedentary women with obesity, and also insisted that improvements in body composition affected positive changes in cardiometabolic parameters. Khairandish et al. [41] reported that no significant changes were found in body composition and blood lipid levels via Pilates intervention for eight weeks, three times a week in sedentary women with obesity. In the present study, we believe that the absence of positive changes in body composition via the Pilates intervention may not have affected changes in cardiometabolic parameters in middle-aged women with obesity [41]. Therefore, to induce positive changes in body composition and cardiometabolic parameters via Pilates intervention, it is necessary to increase the overall exercise volume by increasing the exercise intensity, frequency, time, and period.

Human physiological abilities begin to decline gradually after the age of 30 years, and middle-aged women experience physical and psychological changes more easily than middle-aged men; this includes decreased muscle strength and mass as well as menopause [42,43]. Middle-aged women show reduced physical function (e.g., decreased muscle strength and mass and increased fat mass), and these weakened physical functions lead to muscle shortening, overuse, and unbalanced posture, deteriorating muscle mechanical properties [44]. Various exercise modalities are used to relieve and relax muscle tension, mainly to improve the stability and flexibility of core muscles, and as a program for musculoskeletal pain relief [45]. Cronin et al. [46] reported that muscle tone and stiffness were significantly improved by conducting an online Pilates intervention once a week for six weeks in stroke patients. Dolgion et al. [47] reported that the mechanical properties of muscles were significantly improved as a result of a Pilates intervention for four weeks, twice a week, 60 min a day for college students. Villanueva et al. [48] reported that the mechanical properties of core muscles were significantly improved by conducting a musculoskeletal relaxation exercise program for 15 min daily for 6 weeks in office workers. Abdominal muscle strength, erector spinae muscles, hip joint muscles, and pelvic stability are often weakened in middle-aged women because of a decrease in muscle strength during pregnancy and childbirth, and musculoskeletal disorders increase [49]. In particular, the deep muscles of the back are weak and unbalanced, and their ability to control postural balance is reduced because of a decrease in the proprioceptive sensory function, resulting in body shaking [50]. In the present study, muscle tone and stiffness of the rectus abdominis, external oblique, and erector spinae were measured using Myoton PRO (Myoton AS, Tallinn, Estonia) and were found to be improved by face-to-face and online Pilates intervention for 12-week. These results are believed to have helped stabilize the lumbar region by activating mechanoreceptors located in the muscle tissues around the core and are interpreted as the result of improved physiological functions of the core muscles. In addition, these results were shown to be closely related to improvements in sit-ups and sit-and-reach among physical fitness factors. However, the improvements in muscle tone and stiffness were greater with the FPG than with the OPG. This means that face-to-face Pilates, which involves performing Pilates with more precise movements with an instructor, is a more effective modality than online Pilates for improving core muscle mechanical properties.

Stress and depression are related to changes in the hypothalamic–pituitary–adrenal (HPA) and sympathetic nervous system (SNS) axes and affect factors such as breathing, blood pressure and temperature regulation, behavior, sleep, and physical activity [51]. The HPA axis activates cortisol secretion and the renin–angiotensin system, resulting in problems such as hyperglycemia, inflammation, and hypertension, while the SNS stimulates the secretion of the neurotransmitters epinephrine and norepinephrine, worsening HRV and blood viscosity [52,53,54,55]. Pilates is an exercise that trains core stability, muscle strength, flexibility, breathing, and posture, and is also recommended for improving stress, depression, and anxiety disorders [45]. Pilates is an effective exercise modality for improving mental health because it reduces stress by activating the parasympathetic nervous system (PNS) via autonomous breathing control [36]. Regarding previous studies on Pilates intervention and mental health, Cavina et al. [56] reported that Pilates intervention for 12 weeks, three times a week, and 60 min a day for adult males significantly improved mental health-related cardiac autonomic control variables (e.g., SDNN, LF, and HF). Fleming et al. [24] confirmed that an 8-week Pilates intervention significantly improved mental health-related anxiety and depression. In addition, Akbas and Ünver [57] reported that Pilates intervention for 6 weeks, twice a week, 60 min per day significantly improved various mental health-related parameters such as depression and quality of life in women. In the present study, we measured HRV to analyze changes in ANS activity, which is an important factor in mental health, and evaluated state anxiety and depression using questionnaires. Our study confirmed that both Pilates interventions (face-to-face Pilates and online Pilates) significantly improved mental health parameters in middle-aged women with obesity. These results indicate that regardless of the form of Pilates (e.g., face-to-face or online), Pilates intervention is an effective alternative for enhancing mental health by improving ANS modulation through an increase in PNS activity and a decrease in SNS activity. However, HRV, STAI, and BDI, which are mental health parameters, showed significantly greater improvements in the FPG than in the OPG. These results are because face-to-face Pilates intervention via the instructor significantly improves the modulation of the ANS through precise movement and deep breathing, and because the instructor’s immediate feedback and enthusiasm promote interaction with the participant, which has a positive effect on motivation for exercise and depression [58,59].

Pilates strengthens and trains core muscles by activating the central core of the body and is effective in improving core muscular endurance and flexibility [60,61,62]. Suner-Keklik et al. [23] reported that online Pilates intervention performed on a mat for six weeks in healthy individuals had positive effects on trunk proprioception and core muscle endurance, and they insisted that the contribution of Pilates intervention to the development of both muscular endurance and proprioceptive senses, even if performed at a distance, is important. Park et al. [63] reported that 12 weeks of Pilates core stability intervention effectively improved dynamic balance in archers. Oliveira et al. [64] determined the effects of Pilates intervention on lower leg strength and postural balance in older adults and concluded that Pilates intervention led to significant improvements in isokinetic torque of the knee extensors and flexors and postural balance in older adults. Bullo et al. [65] performed a systematic review to summarize the effects of Pilates intervention on physical fitness in the elderly population and reported that Pilates intervention was an effective modality for improving muscle strength, walking and gait performance, balance, and flexibility. In the present study, we confirmed that face-to-face and online Pilates interventions for 12 weeks were effective for endurance, flexibility of the core muscles, and balance function, and these results were consistent with those of previous studies. However, muscular endurance and flexibility in core muscles and balance function were significantly improved in the FPG compared to the OPG, and these results are because the instructor’s appropriate personal guidance and management were provided in the FPG than in the OPG. Güngör et al. [21] investigated the effects of Pilates intervention based on supervised or home-based core stability on lower extremity strength and postural control and concluded that supervised Pilates intervention is more effective than home-based Pilates intervention in improving strength, postural control, core stability, physical capacity, and fatigue. The results of these previous studies are consistent with those of this study.

Taken together, Pilates intervention using an online platform is easy to access, efficient in time management, and has positive effects; however, face-to-face Pilates intervention via an instructor has a greater positive effect on muscle mechanical properties, mental health, and physical fitness in middle-aged women with obesity due to higher motivation for participation and precise movements.

## 5. Limitations

The present study has several limitations. (1) Participants in the present study were premenopausal middle-aged women with obesity aged 30–50 years (>30% body fat); however, their menstrual cycle and sex hormone concentrations were not measured. (2) The present study did not investigate the amount of physical activity or dietary intake of participants during the intervention period. (3) The normality of the distribution of all outcome parameters was verified using the Shapiro–Wilk W-test prior to the parametric tests; however, the small sample size may limit the interpretation of the results of this study.

## 6. Conclusions

The 12-week face-to-face and online Pilates sessions elicited effective improvements in muscle mechanical properties (e.g., core muscle tone and stiffness), mental health (e.g., HRV, STAI, and BDI), and physical fitness (e.g., muscular endurance and flexibility in core muscles and balance function). However, face-to-face Pilates was confirmed to be a more effective modality for improving health-related functions than online Pilates. In the future, we believe that research will be needed to develop new strategies to increase the effectiveness of online Pilates and compare their effectiveness with face-to-face Pilates.

## Figures and Tables

**Table 1:** Participant characteristics (mean ± standard deviation).

Parameters	FPG	OPG	CG	*p*-Value
Age (year)	43.00 ± 5.56	42.20 ± 5.45	44.60 ± 5.50	0.616
Height (cm)	161.52 ± 5.39	159.43 ± 4.50	160.04 ± 6.00	0.670
Weight (kg)	63.85 ± 9.86	66.05 ± 8.96	68.97 ± 7.60	0.442
BMI (kg/m^2^)	24.47 ± 3.48	25.97 ± 3.15	26.99 ± 3.55	0.266
Free fat mass (kg)	40.55 ± 4.56	40.50 ± 4.17	42.64 ± 3.41	0.419
Body fat mass (kg)	23.33 ± 6.63	25.55 ± 5.60	26.35 ± 5.96	0.524
Percent body fat (%)	36.08 ± 5.31	38.38 ± 3.91	37.80 ± 5.07	0.545

Note. FPG, face-to-face Pilates group; OPG, online Pilates group; CG, control group; BMI, body mass index.

**Table 2:** Body composition (mean ± standard deviation).

Parameters	Groups	Test		∆%	*p* (*η*^2^)	
		Pre	Post			
Weight (kg)	FPG	63.85 ± 9.86	63.34 ± 8.79	−0.80	G	0.319 (0.081)
	OPG	66.05 ± 8.96	65.54 ± 7.22	−0.77	T	0.661 (0.007)
	CON	68.97 ± 7.60	69.42 ± 6.64	0.65	G × T	0.579 (0.040)
BMI (kg/m^2^)	FPG	24.47 ± 3.48	24.30 ± 3.31	−0.72	G	0.194 (0.114)
	OPG	25.97 ± 3.15	25.81 ± 2.86	−0.62	T	0.777 (0.003)
	CON	26.99 ± 3.55	27.18 ± 3.27	0.72	G × T	0.600 (0.037)
Free fat mass (kg)	FPG	40.55 ± 4.56	40.84 ± 4.21	0.72	G	0.461 (0.056)
	OPG	40.50 ± 4.17	40.67 ± 4.09	0.42	T	0.546 (0.014)
	CON	42.64 ± 3.41	42.49 ± 3.23	−0.35	G × T	0.553 (0.043)
SMM (kg)	FPG	21.92 ± 2.71	22.07 ± 2.46	0.68	G	0.442 (0.059)
	OPG	21.98 ± 2.47	22.10 ± 2.43	0.55	T	0.443 (0.022)
	CON	23.21 ± 2.00	23.18 ± 2.02	−0.13	G × T	0.748 (0.021)
Body fat mass (kg)	FPG	23.33 ± 6.63	22.64 ± 6.02	−2.96	G	0.378 (0.070)
	OPG	25.55 ± 5.60	25.07 ± 4.75	−1.88	T	0.428 (0.023)
	CON	26.35 ± 5.96	26.70 ± 5.29	1.33	G × T	0.429 (0.061)
Percent body fat (%)	FPG	36.08 ± 5.31	35.14 ± 4.99	−2.61	G	0.398 (0.066)
	OPG	38.38 ± 3.91	37.77 ± 3.72	−1.59	T	0.187 (0.063)
	CON	37.80 ± 5.07	38.29 ± 4.60	1.30	G × T	0.083 (0.169)
WHR	FPG	0.870 ± 0.043	0.866 ± 0.037	−0.51	G	0.947 (0.004)
	OPG	0.869 ± 0.059	0.867 ± 0.051	−0.22	T	0.815 (0.002)
	CON	0.875 ± 0.083	0.876 ± 0.064	0.13	G × T	0.954 (0.003)

Note. FPG, face-to-face Pilates group; OPG, online Pilates group; CG, control group; BMI, body mass index; SMM, skeletal muscle mass; WHR, waist–hip ratio; G: Group, T, time; G × T, interaction.

**Table 3:** Muscle mechanical property (mean ± standard deviation).

Parameters		Groups	Test		∆%	*p* (*η*^2^)		
			Pre	Post				
Rectus abdominis	Tone (Hz)	FPG	11.68 ± 1.67	9.88 ± 0.80	−15.41 **	G	0.301 (0.085)	
		OPG	11.02 ± 1.30	10.05 ± 0.45	−8.80 *	T	0.002 (0.304)	++
		CON	11.08 ± 1.41	11.35 ± 1.01	2.44	G × T	0.006 (0.313)	++
	Stiffness (N/m)	FPG	172.95 ± 10.40	157.25 ± 16.55	−9.08 *	G	0.340 (0.077)	
		OPG	170.40 ± 15.05	162.10 ± 13.29	−4.87 *	T	0.009 (0.228)	++
		CON	170.90 ± 10.61	172.90 ± 10.62	1.17	G × T	0.032 (0.225)	+
External oblique	Tone (Hz)	FPG	12.50 ± 1.45	11.07 ± 0.97	−11.44 ***	G	0.474 (0.054)	
		OPG	12.41 ± 1.07	11.76 ± 1.01	−5.24 *	T	0.000 (0.421)	+++
		CON	12.22 ± 0.91	12.46 ± 0.94	1.96	G × T	0.000 (0.473)	+++
	Stiffness (N/m)	FPG	186.80 ± 16.28	167.35 ± 19.05	−10.41 **	G	0.507 (0.049)	
		OPG	186.20 ± 18.65	176.10 ± 13.40	−5.42 *	T	0.004 (0.275)	++
		CON	183.95 ± 19.02	186.50 ± 16.39	1.39	G × T	0.013 (0.276)	+
Erector spinae	Tone (Hz)	FPG	16.34 ± 1.69	15.05 ± 1.54	−7.89 **	G	0.687 (0.027)	
		OPG	16.57 ± 1.79	15.89 ± 1.91	−4.10 *	T	0.002 (0.305)	++
		CON	16.10 ± 1.66	16.43 ± 1.54	2.05	G × T	0.001 (0.396)	++
	Stiffness (N/m)	FPG	329.65 ± 57.14	297.45 ± 50.71	−9.77 **	G	0.790 (0.017)	
		OPG	333.85 ± 49.02	312.45 ± 51.01	−6.41 *	T	0.003 (0.289)	++
		CON	323.45 ± 46.59	331.75 ± 30.68	2.57	G × T	0.003 (0.334)	++

Note. FPG, face-to-face Pilates group; OPG, online Pilates group; CG, control group; G: Group, T, time; G × T, interaction. + < 0.05, ++ < 0.01, +++ < 0.001: Significant interaction and/or main effects. * < 0.05, ** < 0.01, *** < 0.001: Significantly different between pre- and post-intervention.

**Table 4:** Cardiometabolic parameters (mean ± standard deviation).

Parameters	Groups	Test		∆%	*p* (*η*^2^)	
		Pre	Post			
SBP (mmHg)	FPG	118.60 ± 10.25	114.05 ± 10.56	−3.84	G	0.560 (0.042)
	OPG	122.85 ± 12.89	119.40 ± 13.96	−2.81	T	0.059 (0.126)
	CON	118.70 ± 7.48	118.15 ± 6.92	−0.46	G × T	0.514 (0.048)
DBP (mmHg)	FPG	75.80 ± 9.01	74.30 ± 7.97	−1.98	G	0.988 (0.001)
	OPG	76.00 ± 11.03	74.60 ± 10.60	−1.84	T	0.293 (0.041)
	CON	74.85 ± 5.53	74.60 ± 5.91	−0.33	G × T	0.847 (0.012)
MAP (mmHg)	FPG	90.06 ± 8.89	87.57 ± 8.45	−2.76	G	0.889 (0.009)
	OPG	91.62 ± 11.25	89.52 ± 10.94	−2.29	T	0.123 (0.086)
	CON	89.46 ± 5.84	89.12 ± 5.99	−0.38	G × T	0.668 (0.029)
TG (mg/dL)	FPG	120.20 ± 66.75	101.10 ± 62.03	−15.89	G	0.730 (0.023)
	OPG	126.60 ± 72.12	113.10 ± 68.35	−10.66	T	0.121 (0.087)
	CON	132.00 ± 50.73	130.80 ± 43.16	−0.91	G × T	0.575 (0.040)
TC (mg/dL)	FPG	194.70 ± 22.42	185.80 ± 24.52	−4.57	G	0.738 (0.022)
	OPG	189.50 ± 56.50	183.30 ± 41.20	−3.27	T	0.438 (0.022)
	CON	197.40 ± 23.01	194.36 ± 24.14	−1.54	G × T	0.953 (0.004)
HDL-C (mg/dL)	FPG	58.50 ± 10.28	65.70 ± 15.46	12.31	G	0.183 (0.118)
	OPG	52.60 ± 13.48	57.30 ± 12.84	8.94	T	0.066 (0.120)
	CON	53.30 ± 8.25	53.60 ± 12.29	0.56	G × T	0.416 (0.063)
LDL-C (mg/dL)	FPG	119.96 ± 34.65	103.06 ± 25.26	−14.09	G	0.921 (0.006)
	OPG	118.28 ± 56.96	105.98 ± 35.23	−10.40	T	0.266 (0.046)
	CON	117.94 ± 25.42	116.35 ± 60.43	−1.35	G × T	0.779 (0.018)
Glucose (mg/dL)	FPG	102.20 ± 6.00	97.88 ± 5.29	−4.22	G	0.113 (0.149)
	OPG	105.72 ± 5.15	103.30 ± 8.31	−2.29	T	0.103 (0.095)
	CON	101.74 ± 5.82	101.30 ± 5.33	−0.44	G × T	0.545 (0.044)

Note. FPG, face-to-face Pilates group; OPG, online Pilates group; CG, control group; SBP, systolic blood pressure; DBP, diastolic blood pressure; MAP, mean arterial pressure; TG, triglyceride; TC, total cholesterol; HDL-C, high-density lipoprotein cholesterol; LDL-C, low-density lipoprotein cholesterol; G: Group, T, time; G × T, interaction.

**Table 5:** Mental health (mean ± standard deviation).

Parameters		Groups	Test		∆%	*p* (*η*^2^)		
			Pre	Post				
HRV	Mean RR (msec)	FPG	741.65 ± 92.26	821.53 ± 84.60	10.77 *	G	0.315 (0.082)	
		OPG	798.64 ± 91.62	850.04 ± 76.97	6.44	T	0.024 (0.174)	+
		CON	837.61 ± 97.36	824.07 ± 91.33	−1.62	G × T	0.077 (0.173)	
	SDNN (ms)	FPG	27.23 ± 10.03	34.78 ± 9.64	27.73 ***	G	0.541 (0.044)	
		OPG	25.41 ± 8.98	30.07 ± 10.94	18.34	T	0.004 (0.269)	++
		CON	33.21 ± 9.73	31.34 ± 9.38	−5.65	G × T	0.005 (0.326)	++
	RMSSD (ms)	FPG	34.58 ± 14.48	41.33 ± 11.67	19.50 *	G	0.771 (0.019)	
		OPG	32.04 ± 18.42	35.75 ± 16.53	11.58 *	T	0.008 (0.233)	++
		CON	39.20 ± 18.43	37.93 ± 15.07	−3.24	G × T	0.017 (0.261)	+
	LF (ms^2^)	FPG	197.12 ± 63.57	164.33 ± 44.10	−16.64	G	0.549 (0.043)	
		OPG	185.26 ± 32.50	166.08 ± 26.18	−10.35	T	0.082 (0.108)	
		CON	192.56 ± 59.25	196.86 ± 42.05	2.23	G × T	0.238 (0.101)	
	HF (ms^2^)	FPG	191.58 ± 48.35	212.52 ± 51.91	10.93	G	0.936 (0.005)	
		OPG	189.71 ± 50.08	201.51 ± 37.92	6.22	T	0.118 (0.088)	
		CON	196.26 ± 42.32	197.56 ± 44.54	0.67	G × T	0.529 (0.046)	
	LF/HF ratio	FPG	1.05 ± 0.26	0.78 ± 0.14	−25.17 *	G	0.421 (0.062)	
		OPG	1.02 ± 0.24	0.84 ± 0.12	−18.38 *	T	0.007 (0.242)	++
		CON	0.98 ± 0.20	1.01 ± 0.17	3.58	G × T	0.041 (0.210)	+
STAI (score)		FPG	41.80 ± 8.75	33.40 ± 4.74	−20.10 **	G	0.634 (0.033)	
		OPG	39.40 ± 9.58	35.50 ± 10.38	−9.90 *	T	0.031 (0.162)	+
		CON	39.20 ± 9.27	41.50 ± 7.55	5.87	G × T	0.020 (0.250)	+
BDI (score)		FPG	12.20 ± 4.64	8.00 ± 5.50	−34.43 *	G	0.376 (0.070)	
		OPG	11.10 ± 5.13	9.10 ± 4.61	−18.02	T	0.058 (0.127)	
		CON	11.80 ± 4.92	13.00 ± 3.59	10.17	G × T	0.046 (0.204)	+

Note. FPG: face-to-face Pilates group; OPG: online Pilates group; CG: control group; HRV: heart rate variability; Mean RR: average R-R interval duration; SDNN: standard deviation of normal to normal interval; RMSSD: root mean square differences of successive R-R intervals; LF: low frequency; HF: high frequency; STAI: state-trait anxiety inventory; BDI: Beck depression inventory; G: Group, T: time; G × T: interaction. + < 0.05, ++ < 0.01: Significant interactions and/or main effects. * < 0.05, ** < 0.01, *** < 0.001: Significantly different between pre- and post-intervention.

**Table 6:** Physical fitness (mean ± standard deviation).

Parameters	Groups	Test		∆%	*p* (*η*^2^)		
		Pre	Post				
Dominant grip strength (kg)	FPG	23.56 ± 3.35	25.97 ± 3.93	10.23	G	0.270 (0.092)	
	OPG	26.55 ± 5.50	28.88 ± 4.76	8.78	T	0.079 (0.110)	
	CON	24.96 ± 7.00	24.17 ± 5.54	−3.17	G × T	0.138 (0.136)	
Non-dominant grip strength (kg)	FPG	21.17 ± 4.67	23.25 ± 5.53	9.83	G	0.111 (0.150)	
	OPG	26.12 ± 5.16	27.79 ± 5.12	6.39	T	0.309 (0.038)	
	CON	24.46 ± 7.58	23.60 ± 4.47	−3.53	G × T	0.386 (0.068)	
Sit-up (number)	FPG	15.60 ± 6.83	17.90 ± 6.66	14.74 **	G	0.666 (0.030)	
	OPG	16.10 ± 7.89	17.50 ± 8.28	8.70 *	T	0.001 (0.360)	++
	CON	14.30 ± 5.81	14.50 ± 4.74	1.40	G × T	0.051 (0.198)	
Sit-and-Reach (cm)	FPG	8.92 ± 8.82	15.49 ± 9.16	73.65 **	G	0.683 (0.028)	
	OPG	9.40 ± 6.18	14.16 ± 8.40	50.64 *	T	0.000 (0.548)	+++
	CON	8.73 ± 10.20	9.35 ± 10.24	7.10	G × T	0.005 (0.322)	++
Right Balance (sec)	FPG	9.09 ± 8.32	18.15 ± 13.14	99.78 *	G	0.249 (0.098)	
	OPG	9.79 ± 8.05	16.02 ± 13.67	63.74	T	0.008 (0.235)	++
	CON	7.62 ± 5.91	7.98 ± 5.75	4.71	G × T	0.155 (0.129)	
Left Balance (sec)	FPG	8.66 ± 8.30	18.15 ± 15.47	109.51 *	G	0.164 (0.125)	
	OPG	9.29 ± 7.06	16.57 ± 16.44	78.40	T	0.014 (0.203)	+
	CON	6.47 ± 5.38	6.63 ± 2.37	2.39	G × T	0.199 (0.113)	
VO_2_max (ml/kg/min)	FPG	25.01 ± 5.06	27.13 ± 7.22	8.48	G	0.316 (0.082)	
	OPG	23.11 ± 4.46	24.18 ± 3.90	4.63	T	0.338 (0.034)	
	CON	23.48 ± 5.30	22.81 ± 3.81	−2.85	G × T	0.422 (0.062)	

Note. FPG, face-to-face Pilates group; OPG, online Pilates group; CG, control group; VO_2_max, maximal oxygen uptake; G: Group, T, time; G × T, interaction. + < 0.05, ++ < 0.01, +++ < 0.001: Significant interaction and/or main effects. * < 0.05, ** < 0.01: Significantly different between pre- and post-intervention.

## Data Availability

The data presented in this study are available upon request from the first or corresponding author.
